# The Impact of Lifestyle Changes on the Physical and Mental Health of Emergency Medicine Staff and Their Association With Well-Being at a Major Tertiary Hospital

**DOI:** 10.7759/cureus.71203

**Published:** 2024-10-10

**Authors:** Mashaeel H Almutairi, Abdulrahman S Albazie, Dina S Al Sufyani

**Affiliations:** 1 Emergency Department, Prince Sultan Military Medical City, Riyadh, SAU

**Keywords:** emergency medicine staff, physical and mental health, prince sultan medical city, the impact of lifestyle changes, well-being

## Abstract

Introduction

The impact of lifestyle changes on the physical and mental health of emergency medicine staff has been a topic of increasing concern due to its effect on healthcare quality. This study aimed to assess the impact of lifestyle changes on emergency medicine staff's physical and mental health and their association with well-being at a major tertiary hospital.

Methods

A cross-sectional study was conducted in December 2023 using an online questionnaire targeting Emergency Department (ED) staff in Prince Sultan Medical City, Riyadh, Saudi Arabia. The data were analyzed using SPSS software (IBM Corp., Armonk, NY), with a Spearman correlation test and simple linear regression analysis used to determine the relationship between variables.

Results

The study included 149 participants, primarily male (59.7%) and predominantly within the age group of 30-39 years (59.1%). The sample consisted of ED nurses, residents, consultants, and paramedics, with over half having more than five years of experience in emergency medicine. Lifestyle changes were evident among participants, with 60.4% reporting worsened sleep patterns, 49.7% engaging in less physical activity, and 56.4% reporting poorer eating habits after joining the ED. Well-being assessments revealed moderate well-being scores (mean = 11.7 ± 5.61), with substantial variability in emotional states. Factors such as night sleep quality, physical activity, and nutrition were significantly correlated with well-being, with night sleep quality showing the strongest positive correlation (rho = 0.349, p < 0.001). Night sleep quality, nutritional intake, and physical activity are significant predictors of well-being, with night sleep quality being the strongest predictor (R^2 ^= 0.122, F = 20.39, p < 0.001).

Conclusion

The study underscores the need for targeted interventions to address lifestyle challenges faced by ED personnel, particularly focusing on improving sleep quality, promoting regular physical activity, and encouraging healthier nutritional habits.

## Introduction

Well-being can be defined as a combination of feeling good and functioning effectively. A healthy lifestyle plays a pivotal role in promoting overall well-being. It is a balance between physical activity, nutritious dietary habits, adequate rest, and mental wellness [1-3]. Maintaining good health contributes to an enhanced quality of life, influencing various dimensions such as physical, educational, emotional, and spiritual well-being [4]. Some habits for maintaining good health include regular exercise and sufficient sleep, which not only contribute to physical fitness but also enhance mood and reduce stress. Adequate sleep is crucial for cognitive function, mood regulation, and overall vitality [3]. Additionally, nurturing mental and emotional well-being through mindfulness practices, stress management, and social connections contributes significantly to a fulfilling life [3,5]. Adopting a positive lifestyle not only fosters longevity but also enhances the quality of life, promoting resilience and the ability to cope with life's challenges [3].

Stress is an inevitable aspect of life, but its impact on well-being underscores the importance of effective coping strategies. Chronic stress has been linked to various health issues, both physical and mental [6]. Prioritizing well-being involves recognizing stressors and implementing coping mechanisms, such as mindfulness, relaxation techniques, and regular physical activity [7,8]. Building a strong support system and fostering social connections also play crucial roles in mitigating the negative effects of stress. Striking a balance between work and personal life, setting realistic goals, and practicing self-compassion contribute to overall well-being [7].

The impact of stress and excessive workloads negatively impact the quality of patient care and the overall health and well-being of healthcare workers (HCWs) [9,10]. Numerous studies convincingly demonstrate that enhancing the well-being of HCWs leads to improved quality of care, increased productivity, and heightened patient satisfaction [9-11]. Emergency department (ED) workers experience higher rates of mental distress and poor health than other HCWs, adversely affecting healthcare quality and safety [9]. The nature of working in an ED often requires healthcare professionals to adapt their lifestyles to effectively cope with the challenges. ED staff frequently experience irregular and demanding work hours, which can significantly impact their daily routines. Shift work, including night shifts, may also disrupt traditional sleep patterns and make it challenging to maintain a consistent and healthy lifestyle [12]. For instance, a study among nurses found a correlation between shift duty and abnormal restraint eating behavior. Nurses on shift duty tended to consume more fast food and snacks while having a lower intake of vegetables [13].

The demanding and unpredictable nature of working in emergency healthcare settings necessitates a closer examination of how professionals in this field adapt their lifestyles to cope with their unique challenges. This study aimed to investigate the lifestyle changes among emergency healthcare providers and examine their impact on overall well-being.

## Materials and methods

Study design

A cross-sectional study was conducted in December 2023 using an online questionnaire. This study targeted ED staff at Prince Sultan Medical City, Riyadh, Saudi Arabia. The questionnaire was designed to capture information on lifestyle changes and well-being.

Study population

The study targeted ED staff, including nurses, residents, consultants, and paramedics. A sample size of 149 participants was achieved, with efforts to ensure a representative sample of various job roles within the ED. Inclusion criteria were ED staff who had been working for at least six months in the department. Exclusion criteria included staff with pre-existing health conditions unrelated to their work environment, as these might confound the results.

Data collection

The questionnaire was distributed along with the invitation to participate through the HCWs' email and social media platforms. The questionnaire was divided into four parts: (1) basic information such as gender, age, marital status, and highest degree; (2) work-related information including job title, years of work in the ED, and monthly night shifts, (3) lifestyle factors such as sleep quality, cigarette consumption, physical activity, weight changes, and nutritional quality, and (4) mental well-being assessed on a Likert scale ranging from 0 (never) to 5 (always).

Before the main study, the questionnaire was pilot-tested with a small subset of ED staff to ensure clarity, relevance, and reliability of the questions. Any feedback has been incorporated into the final version of the questionnaire.

Statistical analysis

Data were analyzed using SPSS software package Version 27.0 (IBM Corp., Armonk, NY). Quantitative data were reported using minimum, maximum, mean, and standard deviation. Categorical and ordinal data were described using numbers and percentages (%). The relationship between ordinal and numerical variables was analyzed using the Spearman correlation test. In addition, a simple linear regression analysis was used to determine each predictor's impact on the dependent variable. Values less than 0.05 were judged to be statistically significant, and values less than 0.01 were considered highly significant.

Ethical considerations

Ethical approval was obtained from the Ethics Committee of Prince Sultan Medical City, Riyadh (IRB Approval No: E-2333). Participants were provided with informed consent forms detailing the purpose of the study, confidentiality of responses, and voluntary participation.

## Results

Table 1 presents the basic demographic characteristics of the study participants. The sample comprised 149 individuals, with a notable gender distribution of 89 (59.7%) males and 60 (40.3%) females. Regarding age distribution, 88 (59.1%) fell in the age group of 30-39 years, followed by those under 30 years (n=49, 32.9%), while participants aged 40 and above collectively represented 8% (n=12) of the sample. Regarding educational attainment, a considerable proportion (n=111, 74.5%) held bachelor's degrees, 30 (20.1%) possessed master's degrees or higher qualifications, and a small percentage (n=8, 5.4%) had diploma-level education. The marital status distribution revealed that slightly over half of the participants (n=78, 52.3%) were married, 67 (45%) were single, and a small fraction (n=4, 2.7%) reported being divorced, separated, or widowed.

**Table 1:** Basic information about the participants in the study

Variables	N	%
Gender		
Female	60	40.3%
Male	89	59.7%
Age (years)		
<30	49	32.9%
30-39	88	59.1%
40-49	6	4.0%
≥50	6	4.0%
Highest education degree		
Diploma	8	5.4%
Bachelor’s degree	111	74.5%
Master’s degree or higher	30	20.1%
Marital status		
Married	78	52.3%
Single	67	45.0%
Divorced/separated/widowed	4	2.7%

Table 2 shows the work-related characteristics of the study participants within the ED. The professional composition of the sample revealed a diverse range of roles; ED nurses constituted the largest group (n=47, 31.5%), followed by Saudi Board of Emergency Medicine (SBEM) residents or service residents (n=40, 26.8%), emergency registrars or consultants (n=31, 20.8%), and emergency staff such as emergency medicine technicians or paramedics (n=26, 17.4%). Notably, 80 (53.7%) had extensive experience, having worked in the ED for over five years, while 58 (38.9%) had one to five years of experience. Regarding night shift frequency, more than half of the participants (n=80, 53.7%) reported working between 5 and 10 night shifts per month, with smaller proportions working either fewer than 5 shifts (n=27, 18.1%) or more than 10 shifts (n=28, 18.8%) monthly.

**Table 2:** Work information of the participants in the study ED, emergency department; EMT, emergency medicine technician; SBEM, Saudi Board of Emergency Medicine; ECG, electrocardiogram; US, ultrasound; CT, computed tomography; MRI, magnetic resonance imaging

Variables	N	%
Job		
ED nurse	47	31.5%
Emergency registrar/consultant	31	20.8%
Emergency staff (EMT/paramedic)	26	17.4%
Medical intern	1	0.7%
SBEM resident/service resident	40	26.8%
Technician (ECG, US, X-ray, CT, and MRI)	4	2.7%
Working years in ED		
<1 year	11	7.4%
1-5 years	58	38.9%
>5 years	80	53.7%
Night shift per month		
0	14	9.4%
<5 night shifts	27	18.1%
5–10 night shifts	80	53.7%
>10 night shifts	28	18.8%

Table 3 presents data on lifestyle changes experienced by ED staff after joining the department. Notably, 60.4% (n=90) of participants reported worsened sleep patterns, including insomnia, since joining the ED. Regarding tobacco consumption, 83 (55.7%) respondents were non-smokers, while 50 (33.6%) reported increased use after joining the ED. Physical activity patterns showed a marked decline, with 74 (49.7%) reporting less physical activity than before joining the ED and 37 (24.8%) reporting no physical activity at all. Weight changes were significant; 62 (41.6%) participants reported weight gain of more than 3 kg. Nutritional habits also saw a decline, with 84 (56.4%) respondents evaluating their eating habits as worse than before joining the ED.

**Table 3:** Lifestyle questions in the study ED, emergency department

Items	N	%
How would you define your night sleep after joining the ED?		
Worsened, I am suffering from insomnia	90	60.4%
My sleep pattern is the same as before	44	29.5%
Improved, I sleep more and better	15	10.1%
Do you think you have increased your tobacco/E-cigarette consumption after joining the ED?		
I do not smoke	83	55.7%
No	16	10.7%
Yes	50	33.6%
Did you exercise before joining the ED?		
No, I was not doing any kind of physical activities	64	43.0%
Yes, 1 hour per week	17	11.4%
Yes, 2-3 hours per week	26	17.4%
Yes, 4 or more hours per week	42	28.2%
In this time, compared to before joining the ED, you can do physical activity		
I don’t do any kind of physical activity	37	24.8%
Less than usual	74	49.7%
Same as before	22	14.8%
More than before	16	10.7%
Has your weight changed after joining the ED?		
I lost 1-2 kg	45	30.2%
My weight has not changed	27	18.1%
I increased 1-2 kg	15	10.1%
I increased >3 kg	62	41.6%
How do you evaluate your quality of nutrition and eating habits compared to before joining the ED?		
Worst	84	56.4%
Same as before	56	37.6%
My eating habits improved	9	6.0%

Table 4 shows data on the well-being and emotional states of ED staff over the past two weeks. The results revealed a varied distribution of responses across the five well-being indicators. For feeling cheerful and in good spirits, the responses were fairly evenly distributed, with slightly higher percentages reporting these feelings "some of the time" (n=40, 26.8%) or "most of the time" (n=34, 22.8%). Similar patterns were observed for feeling calm and relaxed. Notably, for feeling active and vigorous, the highest percentage (n=43, 28.9%) reported this state "less than half of the time." Regarding waking up fresh and rested, a considerable proportion reported this occurring only "some of the time" (n=43, 28.9%) or "less than half of the time" (n=42, 28.2%). Interest in daily life activities showed a relatively even distribution across response categories. The overall well-being score ranged from 2 to 25, with a mean of 11.7 ±5.61 out of a possible maximum score of 25, indicating a moderate level of well-being among the participants during the assessed period.

**Table 4:** The well-being and feeling of the participants in the last two weeks Total well-being possible score=25

Items		At no time	Some of the time	Less than half of the time	More than half of the time	Most of the time	All of the time
I have felt cheerful and in good spirits	N	4	40	33	29	34	9
	%	2.7%	26.8%	22.1%	19.5%	22.8%	6.0%
I have felt calm and relaxed	N	11	40	34	27	29	8
	%	7.4%	26.8%	22.8%	18.1%	19.5%	5.4%
I have felt active and vigorous	N	7	28	43	28	32	11
	%	4.7%	18.8%	28.9%	18.8%	21.5%	7.4%
I woke up feeling fresh and rested	N	14	43	42	25	16	9
	%	9.4%	28.9%	28.2%	16.8%	10.7%	6.0%
My daily life has been filled with things that interest me	N	15	36	34	27	30	7
	%	10.1%	24.2%	22.8%	18.1%	20.1%	4.7%
Total Well-being score	Min -Max		2 - 25	Mean ± SD		11.7 ± 5.61	

Table 5 shows the correlations between various lifestyle factors and overall well-being among ED staff. The results revealed statistically significant positive correlations between three lifestyle factors and well-being scores. Night sleep quality showed the strongest correlation (rho = 0.349, p < 0.001), indicating that better sleep patterns were associated with higher well-being scores. Physical activity levels also demonstrated a significant positive correlation with well-being (rho = 0.189, p = 0.021), suggesting that increased physical activity was linked to improved well-being. Similarly, nutritional intake quality was positively correlated with well-being scores (rho = 0.279, p = 0.001), implying that better eating habits were associated with higher levels of well-being. Conversely, smoking patterns, exercise habits, and weight changes did not show statistically significant correlations with well-being scores in this sample (p > 0.05).

**Table 5:** Correlation between the lifestyle and well-being in the study *Significant at p<0.5. **Highly significant at p<0.1.

Lifestyle items	Spearman correlation	P-value
Night sleep	0.349^**^	0.000
Smoking patterns	0.057	0.494
Exercise	0.049	0.551
Physical activity	0.189^*^	0.021
weight changed	-0.055	0.502
Nutritional intake	0.279^**^	0.001

Table 6 presents the results of the simple linear regression analyses examining the impact of lifestyle changes on well-being among ED staff. The model identified three significant predictors of well-being: night sleep, physical activity, and nutritional intake. Night sleep quality emerged as the strongest predictor, explaining 12.2% of the variance in well-being scores (R^2^ = 0.122, F = 20.39, p < 0.001), with a positive impact (B = 2.90, t = 4.52). Nutritional intake was the second most influential factor, accounting for 7.8% of the variance (R^2^ = 0.078, F = 12.37, p = 0.001), also showing a positive impact (B = 2.56, t = 3.52). Physical activity also showed a significant effect; however, it had a smaller effect, explaining 3.6% of the variance in well-being (R^2^ = 0.036, F = 5.467, p = 0.021), with a positive impact (B = 1.17, t = 2.34). These findings underscore the relative importance of sleep quality, nutrition, and physical activity in predicting well-being among ED staff, with sleep quality demonstrating the most substantial impact.

**Table 6:** Linear regression analysis for the impact of lifestyle changes on the well-being *Significant at p<0.5. **Highly significant at p<0.1. R²: a statistical measure that represents the proportion of the variance in the dependent variable that can be explained by the independent variables in the regression model. Adjusted R²: a modified version of R-squared that adjusts for the number of predictors in the model. F-statistic (F): The F-statistic tests the overall significance of the regression model. B (unstandardized coefficient): This represents the change in the dependent variable for each one-unit change in the corresponding predictor, holding other variables constant. Thus, it shows the direct impact of a predictor on the outcome variable.

Predictors	R^2^	Adjusted R^2^	F	B	Std. error	t	P-value
Night sleep	0.122	0.116	20.39	2.90	0.643	4.52	<0.001**
Physical activity	0.036	0.029	5.467	1.17	0.502	2.34	0.021*
Nutritional intake	0.078	0.071	12.37	2.56	0.727	3.52	0.001**

## Discussion

The study explored the impact of lifestyle changes on emergency medicine staff's physical and mental health and their association with well-being at a major tertiary hospital in Riyadh, Saudi Arabia. The findings show that characteristics such as sleep quality, nutrition, and physical exercise are important in determining the well-being of ED employees.

The study found that night sleep quality was the strongest predictor of well-being among ED staff, accounting for 12.2% of the variation in well-being ratings. This finding is consistent with prior research demonstrating the negative consequences of sleep deprivation on healthcare personnel [8,14]. ED staff work some night shifts as assigned on their rota, which misaligns their natural circadian rhythms, leading to sleep disturbances and negatively affecting both physical and mental health. Shift work, particularly night shifts, disrupts the body’s internal clock, leading to poor sleep quality, increased risk of metabolic disorders, and cognitive impairment [15]. This disruption has a long-term impact on overall well-being and can contribute to the development of chronic conditions such as cardiovascular disease. Moreover, healthcare personnel, particularly those in emergency and critical care settings, frequently work irregular and longer shifts, resulting in sleep problems and higher stress levels [14,15]. Similarly, Tucker et al. found that poor sleep quality is associated with burnout, emotional weariness, and worse job satisfaction among emergency healthcare personnel [16]. Garcia et al. highlighted that burnout among HCWs often leads to increased fatigue, poor sleep, unhealthy eating, and decreased motivation for physical activity [17]. Targeted interventions aiming at promoting sleep hygiene, such as changing work schedules to allow for more consistent sleep patterns, can significantly improve the well-being of ED staff [18]. Mind-body interventions, including mindfulness and yoga, are effective in reducing stress, improving sleep quality, and promoting better physical and mental health outcomes. These practices can help HCWs cope with the demands of their jobs while fostering healthier lifestyle habits [19]. The literature also highlights the importance of cognitive-behavioral therapy for insomnia in reducing sleep problems and increasing mental health outcomes among healthcare professionals [18,20].

Physical exercise was a strong predictor of well-being, consistent with prior research showing that physical activity has a protective effect on mental health [1,3]. A previous meta-analysis found that frequent physical activity is associated with lower levels of depression and anxiety among HCWs [21]. It was found that even moderate physical activity could mitigate the detrimental effects of job-related stress on ED staff [22]. The decrease in physical activity indicated by 49.7% of participants emphasizes the importance of workplace programs that promote physical exercise. Evidence from workplace wellness programs suggests that including short exercise breaks or offering access to on-site fitness facilities can boost physical activity and improve overall well-being [23,24]. This strategy can be especially useful in high-stress settings like emergency rooms.

Nutritional intake quality was the second most powerful factor in predicting well-being, accounting for 7.8% of the variance in well-being ratings. The study found that ED staff's eating habits deteriorated, which is consistent with previous studies showing that HCWs frequently struggle to maintain healthy diets due to tight schedules and restricted access to nutritional meals during shifts [25]. It was found that poor dietary habits among HCWs are connected with increased fatigue, impaired cognitive function, and higher rates of obesity [25]. Increasing access to healthy food alternatives in the workplace, such as providing fresh fruit, vegetables, and balanced meals in hospital cafeterias or vending machines, can have a positive influence on physical and mental health [26]. Furthermore, boosting nutritional education and awareness initiatives for healthcare staff may enhance dietary choices and contribute to better overall health outcomes.

The overall well-being score in the study was moderate, with participants expressing a range of emotional states. This is consistent with previous research indicating that HCWs, particularly those working in high-stress areas such as EDs, frequently experience mood and emotional well-being variations [27]. This association between lifestyle factors (e.g., sleep quality, physical activity, and diet) and emotional well-being emphasizes the significance of taking a comprehensive approach to health that includes both physical and mental aspects. Providing adequate support to ED staff, access to wellness programs, and fostering a culture of self-care could help mitigate the negative effects of lifestyle changes. Previous research found that organizational strategies, such as providing access to wellness programs, promoting a healthy work-life balance, and offering mental health support, are crucial and effective in improving the well-being of HCWs, and organizational support can lead to better management of work-related stress and healthier lifestyle habits, ultimately enhancing well-being [28].

Study limitations

The study has some limitations. Its cross-sectional design limits the ability to establish causal relationships between lifestyle changes and well-being. Self-reported data may introduce biases, including recall bias, as some information asked may have happened a long time ago. This study was conducted in a single tertiary hospital, limiting its generalizability. Additionally, the study did not account for other potential confounding factors such as workload intensity, work-life balance, or access to mental health resources. Moreover, variables such as night sleep were not broken down for sex and ED job, which could have limited the detection of impact depending on sex or roles at the ED (nurses vs. doctors). The sample size of 149 participants may not be large enough to detect all significant associations or allow for robust subgroup analyses. Therefore, addressing these limitations in future research would strengthen the evidence on the impact of lifestyle changes on the well-being of emergency medicine staff.

## Conclusions

The study reveals that sleep quality, physical exercise, and diet significantly impact the overall well-being of ED staff. After joining the department, participants reported negative lifestyle changes, including worsened sleep patterns, decreased physical activity, weight gain, poorer nutritional habits, and increased tobacco smoking. The overall well-being of ED staff was moderate, with sleep quality, nutrition, and physical activity being significant factors. The findings emphasize the need for interventions to address lifestyle determinants, such as adjusting work patterns and schedules, boosting physical exercise, increasing access to good food alternatives, and providing mental health support.
